# Progression independent of relapsing biology in multiple sclerosis: a real-word study

**DOI:** 10.3389/fneur.2025.1595929

**Published:** 2025-05-29

**Authors:** Heather Y. F. Yong, Carlos Camara-Lemarroy

**Affiliations:** ^1^Department of Clinical Neurosciences, University of Calgary, Calgary, AB, Canada; ^2^Cumming School of Medicine, University of Calgary, Calgary, AB, Canada

**Keywords:** progression independent of relapse biology, progression independent of relapse activity, PIRA, multiple sclerosis, MS, relapsing remitting multiple sclerosis, clinically isolated syndrome, real-world

## Abstract

Progression independent of relapse activity (PIRA) implies disability progression in people with relapsing–remitting multiple sclerosis (RRMS) secondary to neurodegeneration. Mechanistically and biologically PIRA could impact the traditional distinction between progressive and relapsing-MS. Herein, we estimated progression independent of relapsing biology (PIRB) in a cohort of 823 participants with clinically-isolated syndrome/RRMS in Calgary, Canada using a modified criterion [excluding relapses, inflammatory MRI activity, interim disability worsening/improvement over the observation period, and progression secondary to alternative causes including formal conversion to secondary-progressive MS]. PIRB was rare and rates remained consistent across disease-modifying therapies (3.75% dimethyl fumarate, 3.67% fingolimod, 3.72% ocrelizumab, 3.52% minocycline) despite varied rates of disability progression. PIRB may offer a practical alternative to the concept of PIRA.

## 1 Introduction

**Figure 1:**
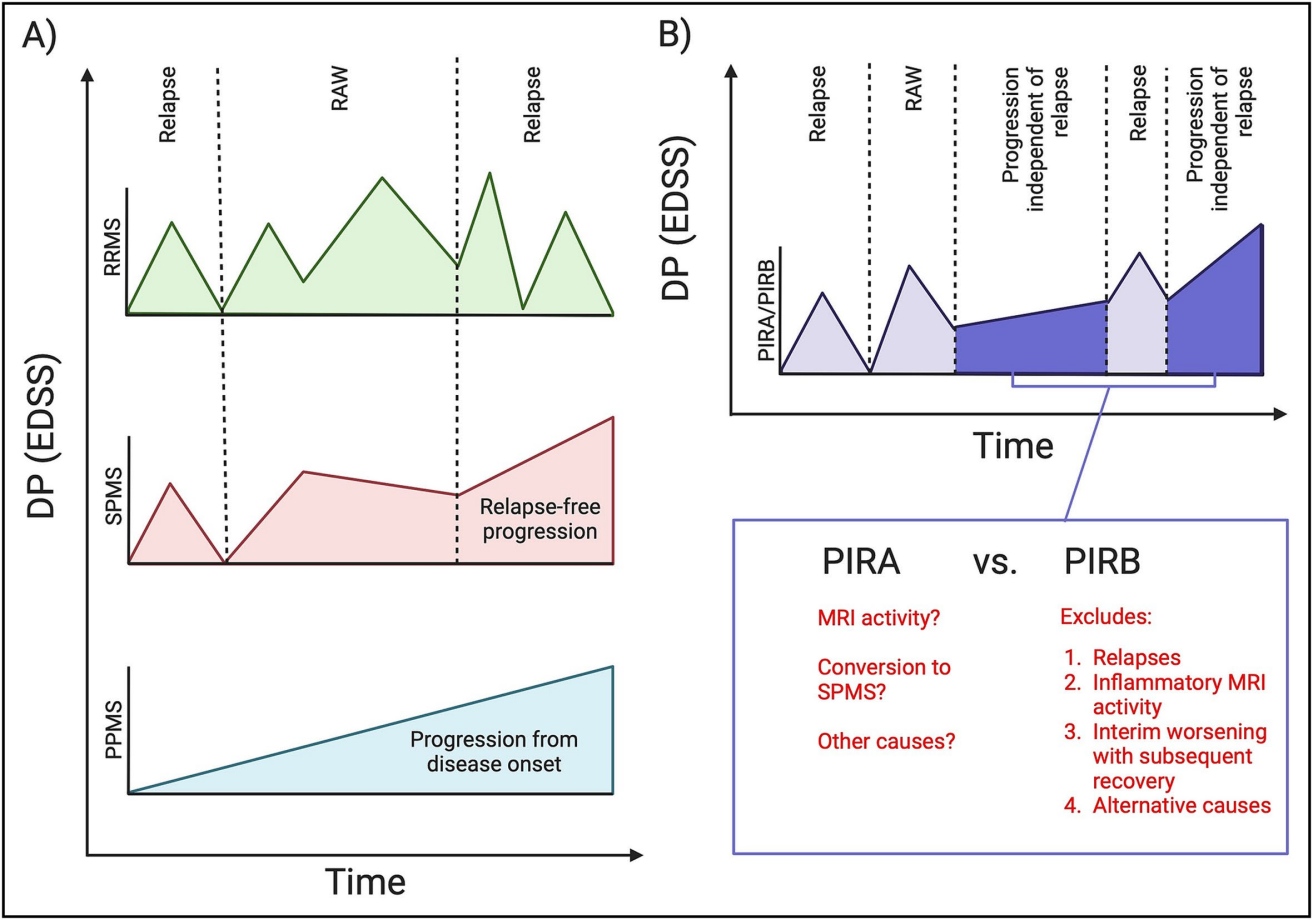
MS disease phenotypes. **(A)** Classically understood MS phenotypes include RRMS (green) which is characterized by relapses and inflammatory MRI activity, with disability progression secondary to relapse-associated worsening (incomplete recovery in between relapses). PPMS (blue) is characterized by progression from disease onset. SPMS (red) is characterized by progression years after an RRMS diagnosis, and in a relapse-free period. **(B)** PIRA/PIRB represents a new concept implying the existence of progression independent of relapses in an RRMS phenotype (dark purple). Both PIRA/PIRB exclude relapse activity, however PIRB also excludes MRI activity, interim worsening/improvement on the EDSS scale, and alternative causes for an EDSS change including medical comorbidities. X-axis = time, Y-axis = disability progression (DP) as measured on the EDSS scale. DP, disability progression; EDSS, expanded disability status scale; PIRA, progression independent of relapsing activity; PIRB, progression independent of relapsing biology; PPMS, primary progressive multiple sclerosis; RAW, relapse associated worsening; RRMS, relapsing–remitting multiple sclerosis; SPMS, secondary progressive multiple sclerosis. Created in BioRender. Yong, HYF (2025) https://BioRender.com/xsuz0zr.

Multiple sclerosis (MS) is an inflammatory and degenerative disease of the central nervous system. Relapsing–remitting MS (RRMS) is characterized by clinical relapses and focal inflammatory demyelination evidenced by MRI (1). Recovery from disability related to relapses is typical, though not universal (relapse-associated worsening with incomplete recovery, RAW). In contrast, progressive MS (PMS) presents as steadily increasing disability secondary to heterogenous neurodegenerative mechanisms (1, 2). PMS is categorized as disability progression (DP) from disease onset (primary progressive MS: PPMS) or years after an RRMS diagnosis (secondary progressive MS: SPMS) (1) (Figure 1A). With the advent of high efficacy disease-modifying therapies (DMTs), a majority of RRMS patients experience disease stability (3), with only modest effects noted in PMS (2).

Recent literature challenges distinct MS phenotypes, suggesting RRMS patients acquire disability through either RAW or progression independent of relapse activity (PIRA) even in the earliest stages of disease (Figure 1B) (4–7). PIRA implies the existence of “progressive biology” in RRMS and is a paradigm shifting concept suggesting MS exists on a continuum with neurodegenerative processes from disease onset. There are several limitations of PIRA that may limit its scope. For example, there is no established PIRA definition impeding comparability amongst studies (5, 6). It also relies on the expanded disability status scale (EDSS) (8) which is highly weighted towards motor disability. Additionally, the extension of PIRA across traditional phenotypes is unclear: the distinction between RAW and PIRA is arbitrarily time-dependent, and a person with RRMS experiencing PIRA after several years could simply be characterized as SPMS. Finally, it does not include evidence of new focal inflammatory disease activity (MRI), which is the closest we have to a biological relapse marker.

In this study, we aimed to isolate progression independent of relapsing biology (PIRB) in a relapsing phenotype using a modified PIRA criterion in both “real-world” and clinical trial MS populations. To do so, we focused on a novel clinical definition applicable to the RRMS population.

## 2 Materials and methods

### 2.1 Study design and participants

Three real-world RRMS cohorts were drawn from the Clinical Impact of Multiple Sclerosis (CIMS) (9), an ongoing observational study of MS patients at the University of Calgary (Alberta, Canada). Participants had started dimethyl fumarate (DMF), fingolimod, or ocrelizumab within 5-years of drug availability (based on provincial regulatory approval). Participants provided written consent, had at least 1 routine follow-up visit during the study period, and could be treatment naïve or have been on other DMTs prior to study initiation. The observation period was 5-years post DMT initiation, although this period was heterogenous due to later ocrelizumab provincial approval.

We included 1 clinical-trial cohort with participants drawn from the phase III minocycline trial in clinically isolated syndrome (Mino (CIS), ClinicalTrials.gov NCT00666887) (10). Key eligibility included a baseline age of 18–60 years old, and CIS within 180 days of trial entry. Patients were randomized (1:1) to either minocycline 100 mg twice daily or placebo. Minocycline was continued until a diagnosis of MS (based on the 2005 McDonald criteria) (11) or 24-months. Methodology and results can be referenced in the original paper (10).

### 2.2 Definitions

Study baseline was defined as the last MS clinic date prior to starting DMF/fingolimod/ocrelizumab, or the protocol-defined minocycline baseline (10). A baseline EDSS was necessary for inclusion. In the University of Calgary MS Clinic, patients are seen annually and an EDSS is obtained by treating neurologists with an MS specialty and EDSS rating certification. MRIs are obtained as part of the usual clinical practice; however, the precise frequency (i.e., annually) varies as does the inclusion of spinal MRI as would be expected in a real-world cohort.

Disability progression was defined as an increase in EDSS by the end of the observation period of ≥1.5 (if baseline EDSS was 0); ≥1 (if baseline EDSS was between 0.5 and ≤5.5); or a ≥ 0.5 increase (if baseline EDSS was >5.5) (5, 12). This EDSS change had to be confirmed/persistent in a visit within 12 months of the end of the observation period. The date of the *first* EDSS change within the observation period was also recorded. Participants were retrospectively registered as PIRB if they met the following criterion (Figure 1B):

(1) DP from baseline to end of study observation period *and*
(2) Absolute exclusion of
  - Clinical relapses at any time during the observation period.
  - Inflammatory MRI activity (new T2 and/or gadolinium enhancing lesions in the brain or spine) at any time during the observation period.
  - Patients who had interim EDSS worsening and improvement over the observation period (commonly seen in relapses), *and*
  - Alternative explanations for an EDSS increase including formal SPMS conversion determined by treating MS specialists, or disability secondary to comorbid medical conditions accounting for the change.

Data from the real-world cohorts was collected at baseline and follow-up (up to 5-years) by 2 separate analysts to retrospectively register patients as those with DP. SPMS conversion was defined according to the treating neurologist diagnosis and extracted if it was formally documented in the medical chart. For those with DP, further data was collected on clinical relapses, MRI activity, or comorbid medical conditions. Data was extracted from 2011 up to November 1st, 2024. In the MinoCIS trial data was obtained as per clinical trial protocol (10).

### 2.3 Outcomes

The primary outcome was the PIRB rate in 3 real-world RRMS cohorts of DMF, fingolimod, and ocrelizumab. Secondary outcomes were the rate of PIRB in a clinical trial environment (MinoCIS) and defining PIRB patient characteristics.

### 2.4 Analysis and descriptive statistics

Baseline characteristics were summarized using frequency (percent) for categorical variables and mean (standard deviation) or median (interquartile range) for continuous variables. Normality of continuous variables was assessed by visual inspection (histograms) and the Shapiro–Wilk test. Based on the normality distribution groups were compared using the Student’s t-test, Mann–Whitney U test, one-way ANOVA, Kruskal-wallis test, or Wilcoxon matched-pairs signed rank test where appropriate. Analysis was done using the Statistical Package GraphPad PRISM (9.0).

### 2.5 Ethics

The CIMS study was approved by the University of Calgary Health Research Ethics Board (REB14-1926) and all participants provided written informed consent. In the MinoCIS study participants provided written informed consent, with relevant institutional review boards/ethics committees approving trial protocols.

## 3 Results

### 3.1 The presence of PIRB in real world cohorts

**Figure 2:**
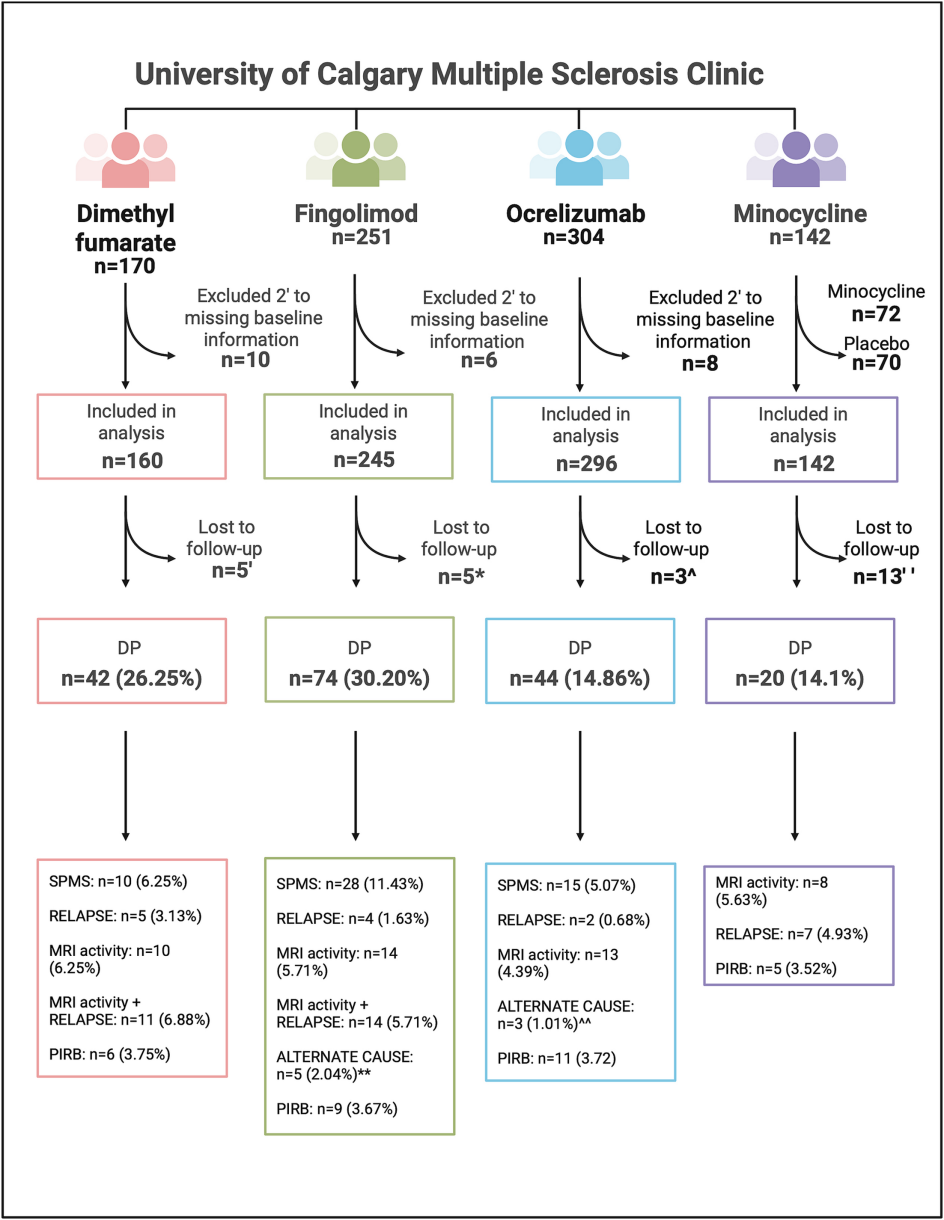
Flowchart of patients included in study analyses, including the breakdown of those who experienced disability progression in the dimethyl fumarate, fingolimod, ocrelizumab, and minocycline cohorts. DP, Disability progression; MRI activity, magnetic resonance imaging inflammatory activity; n, number; PIRB, progression independent of relapsing biology; SPMS, secondary progressive multiple sclerosis. ‘Loss to follow-up in the DMF cohort: *n* = 3 moved away, *n* = 2 unknown. *Loss to follow-up in the fingolimod cohort: *n* = 1 moved away, *n* = 2 deceased, *n* = 2 unknown. **alternative causes for EDSS change in the fingolimod cohort: *n* = 2 stroke, *n* = 1 chemotherapy side effect, *n* = 1 left knee fracture, *n* = 1 psychiatric medication overdose. ^Loss to follow-up in the ocrelizumab cohort: *n* = 1 moved away, *n* = 2 unknown. ^^alternative causes for EDSS change in the ocrelizumab cohort: *n* = 1 compressive myelopathy independent of MS, *n* = 1 severe bilateral median neuropathy, *n* = 1 DMT discontinuation. “Loss to follow-up in the minocycline cohort: *n* = 13 discontinued the study drug or placebo but continued clinical follow-up. Created in BioRender. Yong, HYF (2025) https://BioRender.com/tmdbtlr.

After excluding those without CIMS consent and missing baseline information 701 RRMS participants were included in this study (*n* = 160 DMF, *n* = 245 fingolimod, *n* = 296 ocrelizumab); in total, 13 participants were lost to follow-up and excluded from analysis (Figure 2). Baseline characteristics are summarized in Table 1.

**Table 1:** Baseline participant characteristics in the dimethyl fumarate, fingolimod and ocrelizumab cohorts.

Characteristics	Dimethyl fumarate (*n* = 160)	Fingolimod (*n* = 245)	Ocrelizumab (*n* = 296)	Between group *p* value*
Age at disease onset– mean yr. ± SD	31.03 ± 8.71	31.40 ± 9.05	31.35 ± 8.64	NS
Disease duration at baseline– mean yr. ± SD	11.57 ± 8.84	12.09 ± 7.01	10.52 ± 7.88	0.0137**
Female sex– no. (%)	120 (75.00)	188 (76.42)	220 (74.30)	NS
Baseline EDSS score– median (range)	2 (0–8.0)	2 (0–8.5)	2 (0–6.5)	NS
Baseline treatment naïve– no. (%)	38 (23.75)	51 (20.64)	81 (27.36)	NS
Number of previous DMTs– median (range)	1 (0–4)	1 (0–4)	1 (0–6)	0.0012***

* *p* value for the between-group comparisons of DMF, fingolimod and ocrelizumab using the Kruskal-Wallis test. ** Ocrelizumab versus fingolimod cohort. *** Ocrelizumab versus fingolimod/DMF cohort. DMT, disease modifying therapy; EDSS, expanded disability status scale, n/No., number; NS, not significant; SD, standard deviation; yr, year.

#### 3.1.1 Dimethyl fumarate

**Figure 3:**
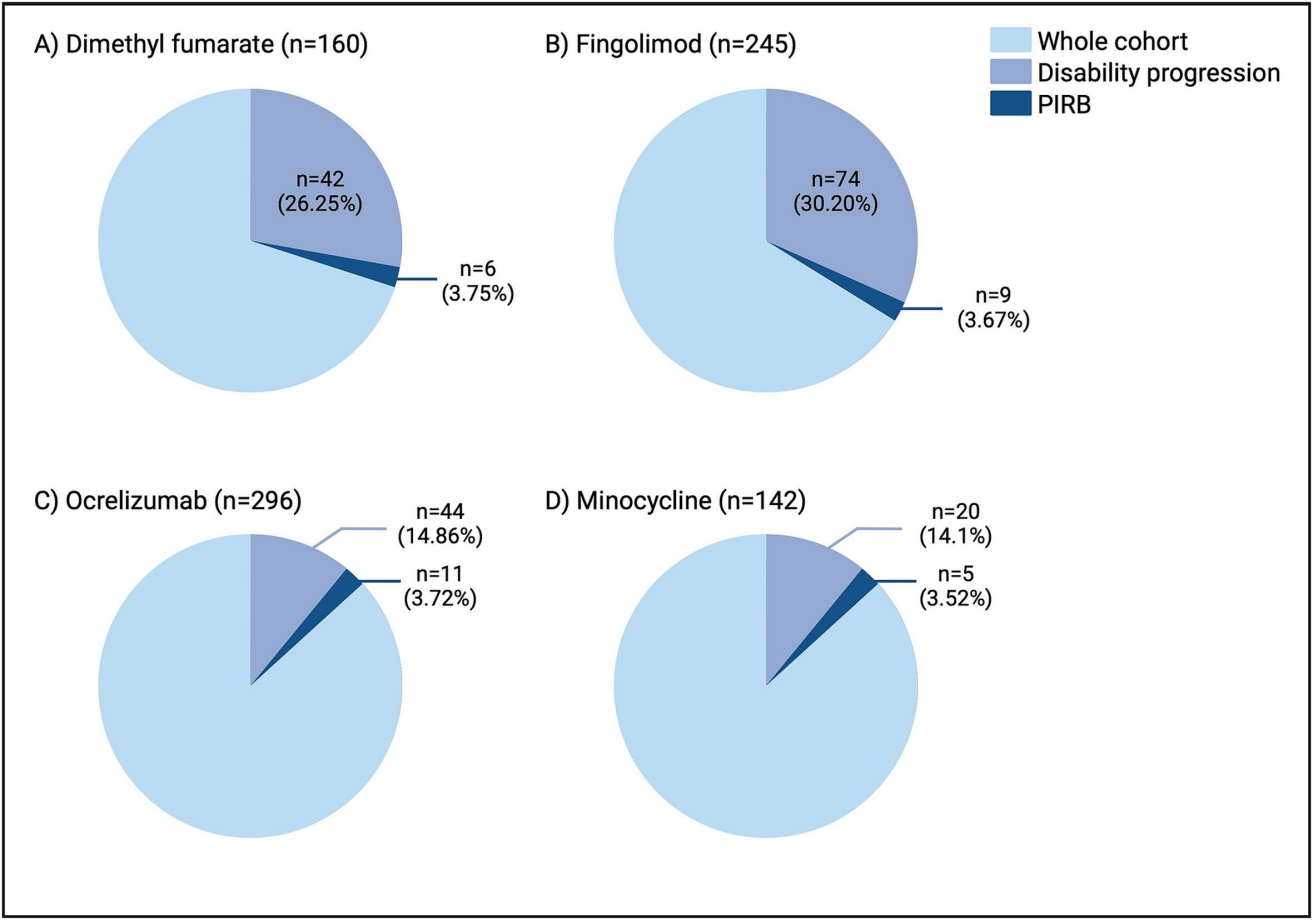
Rates of disability progression and PIRB in the dimethyl fumarate **(A)**, fingolimod **(B)**, ocrelizumab **(C)**, and minocycline **(D)** cohorts. As expected, the rates of disability progression varied amongst the disease-modifying therapies, however the rates of PIRB remained consistent from 3.52–3.75%. n, number; PIRB, progression independent of relapsing biology.

Between 2013 and 2021 (mean follow up time 5.21 years), 42 DMF participants (26.25%) experienced DP. During the observation period, participants had a median of 3 (range 2–8) MRI scans performed (approximately at yearly intervals), and 61.9% of participants had at least one MRI including the spinal cord. Relapses and/or MRI activity accounted for the majority of DP (26 out of 42, 61.9%), followed by formal conversion to SPMS (Figure 2; Table 2). In 23 out of 26 (88.5%) cases the first EDSS change leading to DP occurred within 1 year of relapsing biology events (relapses, new MRI activity, or both); conversely, DP occurred >1 year removed from MRI activity in 2 participants and from both MRI/relapses in 1 participant (Supplementary Table 1). In those with formal conversion to SPMS (*n* = 10, 6.25% of the entire cohort), 50% had either new MRI lesions or a relapse during the observation period. Six participants fulfilled criterion for PIRB accounting for 3.75% of the DMF cohort and 14.29% of those with DP (Figure 3).

**Table 2:** Participant characteristics and follow-up in the dimethyl fumarate, fingolimod and ocrelizumab cohorts for those who experienced disability progression and progression independent of relapsing biology.

Characteristics	Dimethyl fumarate (*n* = 160) DP cohort (*n* = 42, 26.25%) PIRB cohort (*n* = 6, 3.75%)			Fingolimod (*n* = 245) DP cohort (*n* = 74, 30.20%) PIRB cohort (*n* = 9, 3.67%)			Ocrelizumab cohort (*n* = 296) DP cohort (*n* = 44, 14.86%) PIRB cohort (*n* = 11, 3.82%)			BG *p* value*
	No.	DP/PIRB vs. entire cohort	DP vs. PIRB	No.	DP/PIRB vs. entire cohort	DP vs. PIRB	No.	DP/PIRB vs. entire cohort	DP vs. PIRB	
Age at disease onset–mean yr. ± SD										
Entire cohort	31.03 ± 8.7130.			31.40 ± 9.05			31.35 ± 8.64			NS
DP cohort	30.22 ± 8.91	NS	NS	30.86 ± 10.39	NS	0.024	30.32 ± 9.13	NS	NS	NS
PIRB cohort	34.26 ± 7.25	NS		37.03 ± 5.18	0.024		29.47 ± 10.49	NS		NS
Disease duration at baseline- mean yr. ± SD										
Entire cohort	11.57 ± 8.84			12.09 ± 7.01			10.52 ± 7.88			0.0137**
DP cohort	10.71 ± 6.88	NS	NS	13.76 ± 7.49	NS	NS	14.82 ± 8.51	0.001	NS	NS
PIRB cohort	11.41 ± 6.66	NS		12.91 ± 7.62	NS		13.87 ± 11.30	NS		NS
Female sex– No. (%)										
Entire cohort	120 (75.00)			188 (76.42)			220 (74.30)			NA
DP cohort	31 (73.81)	NA	NA	50 (65.79)	NA	NA	32 (72.73)	NA	NA	NA
PIRB cohort	6 (100)	NA		6 (66.67)	NA		10 (90.91)	NA		NA
Baseline EDSS score–median (range)										
Entire cohort	2 (0–8)			2 (0–8.5)			2 (0–6.5)			NS
DP cohort	2 (0–6)	NS	NS	2 (0–7.5)	NS	NS	3 (0–6.5)	0.038	0.032	NS
PIRB cohort	2 (0–4)	NS		1.5 (0–5)	NS		1.5 (0–4)	NS		NS
Follow up– mean yr. ± SD										
Entire cohort	5.21 ± 0.50			5.27 ± 0.59			3.36 ± 1.5			<0.0001**
DP cohort	5.15 ± 0.55	NS	NS	5.20 ± 0.65	NS	NS	4.10 ± 1.80	0.007	NS	<0.0001**
PIRB cohort	5.38 ± 0.81	NS		5.08 ± 0.44	NS		4.03 ± 2.04	NS		0.0217**
Follow-up EDSS–median (range)										
Entire cohort	2 (0–8)			2.5 (0–10)’			2 (0–8.5)			0.0002***
DP cohort	4 (1.5–6.5)	<0.0001	NS	5 (1.5–10)	<0.0001	NS	6 (1.5–8.5)	<0.0001	0.01	<0.0001^
PIRB cohort	3 (2–6.5)	0.02		3 (1.5–6.5)	NS		2.5 (1.5–6)	0.04		NS
Progression categorization–No. (% of the entire cohort, % of those with DP)										
SPMS	10 (6.25, 23.81)			28 (11.43, 37.84)			15 (5.07, 34.09)			NA
Relapse	5 (3.13, 11.90)			4 (1.63, 5.45)			2 (0.68, 4.55)			NA
MRI activity	10 (6.25, 23.81)			14 (5.71, 18.92)			13 (4.39, 29.55)			NA
MRI + relapse activity row	11 (6.88, 26.19)			14 (5.71, 18.92)			0			NA
Alternative causes	0			5 (2.04, 5.6.76)”			3 (1.01, 6.82)”’			NA
PIRB	6 (3.75, 14.29)			9 (3.67, 12.16)			11 (3.72, 25.00)			NA

* *p* value for the between-group (BG) comparisons of dimethyl fumarate vs. fingolimod vs. ocrelizumab. ** Ocrelizumab vs. other cohorts. *** Fingolimod vs. other cohorts. ^ Ocrelizumab vs. fingolimod vs. dimethyl (significant differences across all groups). ‘One death secondary to rapid neurologic decline with marked increase in MRI activity. Categorized as having progressed, with relapse + MRI activity. “One participant with predominantly sensory domain changes was diagnosed with peripheral neuropathy secondary to chemotherapy, two participants with pyramidal/cerebellar changes had strokes that accounted for their symptoms, one participant experienced pyramidal changes following a left knee fracture, and one participant experienced cerebral changes secondary to a psychiatric medication overdose. “‘One participant with predominantly pyramidal/sensory domain changes was diagnosed with compressive myelopathy, one participant with sensory domain changes had severe bilateral median neuropathy, and one highly active participant with pyramidal/cerebellar changes discontinued medication. BG, between groups; DP, disability progression; EDSS, expanded disability status scale, MRI, magnetic resonance imaging; n/No., number; NA, not applicable, NS, not significant; PIRB, progression independent of relapsing biology; SD, standard deviation; SPMS, secondary progressive multiple sclerosis; yr, year.

In keeping with our criterion, when compared to the entire DMF cohort EDSS at follow-up was significantly higher in the progressive group (median 4, range 1.5–5.4; *p* < 0.0001) for a change of [+]2 (range 0.5–4). A similar progression was seen in the PIRB cohort with a follow-up EDSS of 3 (range 2–6.5; *p* = 0.0236) and a change of [+]2 (range 1.5–4). Within the DMF population, 100% of PIRB participants were female compared to 75% (entire DMF cohort) and 73.81% (DP cohort). PIRB participants trended towards older age at disease onset that did not reach significance.

#### 3.1.2 Fingolimod cohort

Between 2011 and 2022 (mean follow up time 5.27 years), 74 patients experienced DP (30.20%) with the majority demonstrating formal conversion to SPMS or inflammatory MRI activity (Figure 2; Table 2). Participants had a median of 3 (range 0–8) MRI scans performed, and 43.2% had at least one MRI of the spine. Similarly to the DMF cohort, relapses and/or MRI activity accounted for the majority of DP; in 24 out of 32 (75%) of these cases the first EDSS change leading to defined DP occurred within 1 year of such relapsing biology events. DP occurred >1 year removed from MRI activity in 3 participants, from both MRI/relapses in 3 participants and from relapses in 2 participants (Supplementary Table 1). In those who converted to SPMS (*n* = 28, 11.43% of the entire cohort), 39.3% had either new MRI lesions or a relapse during the observation period. Nine participants (3.67%) fulfilled PIRB criterion (12.16% of those with progression) (Figure 3).

The median follow-up EDSS was higher in the progressive cohort compared to the entire cohort (EDSS 5, range 1.5–10, *p* < 0.0001) for a change of [+]2 (range 0.5–5). The median follow-up EDSS in the PIRB group was 3 (range 1.5–6.5), for a change of [+]1 (range 1–2.5). Similarly to DMF, PIRB participants were older at disease onset when compared to the entire cohort (*p* = 0.024) and those with DP (*p* = 0.024) (Table 2). PIRB participants trended towards a longer disease duration than the entire cohort.

#### 3.1.3 Ocrelizumab cohort

Between 2015 and 2024 (mean follow up time 3.36 years), 44 (14.86%) participants experienced DP. Formal SPMS conversion accounted for the largest group (*n* = 15, 5.07% of the entire cohort, 34.09% of those with DP) (Figure 2; Table 2). Participants had a median of 3 (range 0–5) MRI scans performed, and 56.8% had at least one MRI of the spine. In 8 out of the 15 (53.3%) cases with relapses/MRI activity and DP, the first EDSS change that led to defined DP occurred within 1-year of relapsing biology. DP occurred >1 year removed from MRI activity in 6 participants and from relapses in 1 participant (Supplementary Table 1). In those who converted to SPMS, 33.3% had either new MRI lesions or a relapse during the observation period. Participants who progressed had a longer disease duration (*p* = 0.0014) and baseline EDSS (*p* = 0.038) compared to those who were stable (Table 2). There were lower numbers of relapses (4.55% of those with DP) and MRI activity (29.55% of those with DP) in the ocrelizumab group compared to both DMF/fingolimod. Eleven patients were registered as PIRB (3.72% of the entire cohort, 25% of those who progressed) (Figure 3).

The 5-year EDSS was higher in the progressive group (median 6, range 1.5–8.5) compared to the entire (median 2.0 (range 0–8.5), *p* < 0.0001) and PIRB (median 2.5 (range 1.5–6), *p* = 0.01) cohorts. The 5-year median EDSS change in the PIRB cohort was [+]1.5 (range 1–3). PIRB participants were predominantly female (90.91%) with a trend towards longer disease duration when compared to the entire cohort (Table 2).

#### 3.1.4 Comparisons between cohorts

Between-group (DMF, fingolimod, ocrelizumab) comparisons can be found in Tables 1, 2. Ocrelizumab participants had a shorter follow-up time compared to DMF and fingolimod (*p* < 0.0001) which was expected given the later provincial approval for ocrelizumab. A larger percentage of participants experienced progression in the DMF and fingolimod cohorts compared to ocrelizumab (Figure 3). Primary EDSS domains affected were pyramidal > sensory, cerebellar. Despite differences in progression, the rate of PIRB remained consistent across groups (3.75% for DMF, 3.67% for fingolimod, and 3.72% for ocrelizumab). All groups had a similar number of MRI scans (and rates of spinal cord imaging) during the observation period.

The majority of DP participants were excluded from the PIRB definition due to subclinical MRI activity and SPMS diagnosis. Approximately 61.9% (DMF) and 43.24% (fingolimod) experienced relapse and/or MRI activity, compared to 34.1% in the ocrelizumab cohort. This is in keeping with the presumed higher efficacy of ocrelizumab in RRMS (3). Across all cohorts, those with relapse/MRI activity were younger at disease onset compared to all participants (*p* = 0.0002) and those with DP (*p* = 0.003), a well-known risk factor for relapsing inflammatory activity (13). In contrast, participants with PIRB tended to be female, older at disease onset (*p* = 0.029 compared to all participants; *p* = 0.0079 compared to those with progression) and trended towards a longer disease duration. Finally, the rates of SPMS were higher in the ocrelizumab (34.09% of those with DP) and fingolimod (37.84% of those with DP) cohorts compared to DMF (23.81%) likely reflecting the fact that fingolimod/ocrelizumab DP cohorts were slightly older with a longer disease duration (Table 2). SPMS participants tended to be older at disease onset (NS vs. all participants, *p* = 0.007 vs. those with DP), and had a longer disease duration/higher baseline EDSS (*p* < 0.0001 compared to all participants and those with progression). This is in keeping with known risk factors for SPMS (14).

### 3.2 The presence of PIRB in a clinical trial environment

Utilizing data from the MinoCIS trial 142 participants were included in the analysis (Figure 3). Please refer to the original paper for full demographic data (10). Twenty participants experienced progression during the study period (14.1% of the entire cohort, Figure 3) with the majority experiencing relapses and/or MRI activity (Figure 2; Table 2). Five participants met PIRB criteria (3.52% of the entire cohort, 25% of those with DP), consistent with the real-world cohort rates. There were no significant differences in demographics between the progressive and PIRB groups (not shown).

## 4 Discussion

### 4.1 PIRA in multiple sclerosis

PIRA was introduced to explain the phenomena of RRMS patients experiencing DP in the absence of measurable clinical relapses. The concept has broad implications suggesting MS exists on a continuum with neurodegeneration (progressive biology) superimposed on progression from relapse-associated worsening (relapse biology) (4, 7). PIRA aims to isolate progression from relapsing biology but has been difficult to define. The most recent harmonized definition of PIRA is an increased EDSS score at least 3-months after (and 1-month before) investigator-reported relapse onset (5). Recent evidence suggests that this definition of PIRA does not reliably distinguish between DP due to inflammatory disease activity or neurodegeneration in RRMS (15).

In addition to the challenges in implementing PIRA discussed above, the pathologic correlates remain unknown. PIRA may be driven by the same mechanistic processes as PMS including leptomeningeal inflammation, microglia activity (“compartmentalized inflammation”), and focal brain/spinal cord pathology (5). These speculations are only meaningful if PIRA and relapsing biology are untangled in the first place. Hence, some experts advocate for “relapsing” or “progressive” *biology* instead of distinct clinical phenotypes (16) which was the focus of our study.

### 4.2 Defining PIRB

The following evidence-based justification was utilized to create the PIRB criterion:

(A) *Exclusion of any relapses throughout the observation period*: the terms PIRA/PIRB inherently exclude the presence of clinical relapses. It is important to note that determination of relapse (s) remains the clinical judgement of the treating physician, with inherent bias across patients (17). False positive PIRA events may also be reported if relapses affect unusual domains (ex. cognition or fatigue) (5). PIRA definitions typically account for a time period before/after a relapse in which to assess for progression (as early as 1–3 months after which resolution of symptoms is thought to be unlikely) (4–7). This relatively short interval is likely not adequate in “disconnecting” DP from a preceding relapse, as reversibility/recovery from relapses can take up to a year (6, 18–20). Another goal of exclusion of clinical relapses throughout the observation period was to eliminate EDSS fluctuations secondary to relapse, or relapse-associated worsening; entities that we argue are more evident in “relapsing biology.” Exclusion of clinical relapses temporally removed from DP events (>1 year) may be an overly restrictive criterion, but this only applied to a total of 3 cases in our study (Supplementary Table 1).
(B) *Exclusion of inflammatory MRI activity throughout the observation period*: to further isolate progressive and relapsing biology, we defined inflammatory MRI activity as an indicator of relapsing biology. If MRI lesions are symptomatic, they represent relapses. But even if silent, their underlying biology is that of a relapsing phenotype (as a consequence of inflammatory demyelination) (21, 22) and are more frequent in RRMS with exquisite sensitivity to DMTs (13). Excluding MRI activity has been termed “pure progression,” “pure PIRA” (23), or “progression independent of relapse and MRI activity” (PIRMA) (6) and consistently reduces PIRA rates by ~50% (6, 23–25). In our study, MRI activity in the absence of relapse represented a large proportion of patients that progressed (Figure 2; Table 2). Limitations of PIRMA include a “necessity” for regular spinal cord imaging (which is not done in practice regularly), and that it does not take into account the presence of chronic active lesions (paramagnetic rim and slowly expanding lesions) (6). Moreover, timing of MRI activity and DP is also heterogeneous. Multi-year T2 lesion accumulation can eventually lead to EDSS changes (26, 27), but the underlying biology, according to our definition, would still be “relapsing.” In this study DP events and the timing of relapses and/or new MRI activity were in close temporal association (within a year) in the majority of participants across cohorts. This suggests that “subclinical” focal MRI inflammatory activity is one of the main confounding factors when attempting to isolate relapsing biology. This is similar to the findings in a recent large real-world study on PIRA, where MRI activity led to the re-classification of ~75% of cases (23).
(C) *Exclusion of patients who had any interval worsening in EDSS, with improvement by the end of the observation period*: EDSS is known to be variable, and changes can start from a temporary/“meaningless” drop (18). For example, a 1-point increase in the EDSS due to ipsilateral trigeminal numbness would intuitively indicate a new focal brainstem lesion; however, based on current PIRA definitions this could indicate “progression” instead. Additionally, inter-rater variability, particularly in the lower EDSS sections can fluctuate, can falsely indicate DP that resolves with a different scorer. Moreover, EDSS improvement in SPMS populations is very rare, unless they have had recent relapses (28). Finally, EDSS variability may simply indicate features of the scale itself, now established to be “noisy” (15, 18). By focusing only on confirmed EDSS change at the end of the observation period, we excluded any EDSS variability that may have occurred until then.
(D) *Exclusion of patients with alternative causes for their DP including a formal SPMS diagnosis, and medical conditions that could account for domain changes*: The diagnosis of SPMS relies primarily on physician expertise and can lead to diagnostic delay (in up to 70% of patients) (29). However, the diagnosis of PIRA itself contradicts a diagnosis of SPMS or may simply represent earlier detection of SPMS (30). In our study, our focus was on identifying progressive events in RRMS, and consequently, SPMS diagnosis represented the largest single exclusionary group in both the fingolimod/ocrelizumab cohorts, and second largest for DMF. This raises the question of whether PIRB represents a separate disease phenotype, or is simply selecting for SPMS patients that neurologists are reluctant to classify (secondary to lack of clear definitions, lack of DMTs, or a relatively low EDSS score) (17, 23). More recent definitions of SPMS suggest the requirement of a minimum EDSS of 4.0 (12). Interestingly, 94.3% of all participants in our study who converted to SPMS had a minimum EDSS of 4.0 at last assessment, compared to only 50% of participants who had relapses and/or MRI activity, and 34.6% of those with PIRB. Furthermore, the biological sex of our PIRB population was 84.6% female, compared to 54.7% in those who converted to SPMS, again highlighting a potential biological difference. Moreover, a significant number of participants classified as SPMS had new MRI activity and/or relapse during the observation period as described above (50% in the DMF cohort, 39.3% in the fingolimod cohort, and 33.3% in the ocrelizumab cohort); this again highlights the difficulty in separating biology from clinical phenotypes. Finally, participants in our study were also excluded if they had concurrent comorbidities that could account for a change in their EDSS. Although this is an explicit exclusionary EDSS criterion, it may be overlooked in a small number of cases (8).

### 4.3 PIRB was consistent across cohorts despite varied rates of progression

In this study, we found variability of progression (and DP causes) in the DMF, fingolimod, ocrelizumab, and minocycline cohorts (25.25% vs. 30.20% vs. 14.86% vs. 14.1%) respectively. This result is intuitive, as ocrelizumab is thought to be more efficacious in reducing relapses or MRI activity (3). Another explanation is the shorter follow-up time in both the ocrelizumab (3.36 ± 1.5 years) and minocycline groups (24-months) compared to the fingolimod/DMF cohorts. Despite this variability the rates of PIRB were surprisingly consistent at 3.75% (DMF), 3.67% (fingolimod), 3.82% (ocrelizumab), and 3.52% (minocycline). The uniformity of is similar to what is seen in related literature, with a recent RRMS study showing PIRMA rates of 4.5 and 5.3% at 2 and 3 years (31). Another study of 1,000 MS patients (that notably did not include MRI activity), found that PIRA rates were 3.9–4.2% (32) similar to the rates we have described herein. Importantly, our real-world results are consistent with recent evidence obtained from RRMS populations enrolled in clinical trials, where approximately 4.6% had PIRA independent of MRI inflammatory activity (15). In this study, the “noisy” features of EDSS measurement, and confounders such as transient improvement were also highlighted as problematic. These rates, including those obtained with our PIRB definition, stand in contrast with rates of PIRA in the literature ranging from 4 to 24% (6, 23) and up to >80% (7). Lastly, PIRB participants tended to be older and have a longer disease duration compared to stable participants. Age is known to be strongest unmodifiable risk factor associated with progressive biology (33) in support of our PIRB framework which aimed to isolate relapsing biology.

### 4.4 Clinical implications

As discussed above, the pathological correlates of PIRA (and PIRB) remain uncertain and consistent biomarkers reflecting them have yet to be discovered (34). Using the PIRB definition could represent an opportunity to investigate proposed biomarkers of progressive biology in RRMS. This biological uncertainty translates to DMT use in PIRA/PIRB. Several observational studies support the benefit of DMTs on slowing DP (4, 23, 32, 35, 36) (which could be interpreted as a “residual” effect of relapsing biology where DMT effectiveness is unquestioned), while a number of studies fail to confirm DMT effect in PIRA (31, 37, 38). We argue that PIRB could help avoid this problem and represent a practical alternative biological framework to PIRA. On the other hand, our definition of PIRB could be criticized as being overly narrow/restrictive, but this is a purposeful distinction in response to the broader definition of PIRA. The consistent (and small) rate of PIRB across cohorts suggests that PIRB is not modified by current treatments, independent of their efficacy. Clearly, biological based definitions and phenotypic descriptors urgently need harmonization.

### 4.5 Study limitations

This study had relatively large patient numbers and follow-up times. The main limitations emerge from the observational nature of the study and use of real-world cohorts, although we also included a cohort from a phase III clinical trial (MinoCIS). Participants were heterogenous, with variable ages/DMTs/MRIs available for analysis. The definition for SPMS conversion was clinical, dependent on the judgement of the treating MS neurologist. However, retrospectively defined SPMS as a history of gradual progression after an initial RRMS course is still the most commonly used criteria in the clinic (1). Thus, these limitations are an accurate reflection of real-world practice. The assessment of PIRB in populations under different DMTs could also add bias, given different follow-up times or diagnostic criteria at the time of treatment. However, the fact that the rates of PIRB were consistent across cohorts could also be viewed as a strength of our proposed framework. Longer observation times could also impact on our findings. A recent real-world study found that most DP events may occur past the 5-year mark in RRMS (23).

Additionally, our study relied heavily on the EDSS scale. The relatively low median EDSS at last observation in the PIRB groups (ranging from 2.0–3.0) could indicate that EDSS “noise” may be a confounder, and longer follow-up periods may still show reversibility of DP. With the application of more sensitive clinical criteria, the ability to detect PIRB may be greater. Further, while we felt our study may indicate the presence of “progressive biology” in some individuals with RRMS, our data did not inform on the presence of postulated correlates of progressive biology such as brain atrophy, paramagnetic rim lesions or slowly-expanding lesions (2). While the MS field lacks pure “progressive” biology biomarkers, PIRB remains clinically applicable. Future studies should validate the consistency of PIRB and moving towards a cohesive (and inclusive) definition of PIRA/PIRMA/PIRB.

## 5 Conclusion

PIRA has implications for our understanding of MS biology, clinical practice, and DMT use. Using a more robust definition that we termed progression independent of relapsing *biology*, we demonstrated that a minority of RRMS patients experience PIRB (3.52–3.75%). PIRB may represent a practical alternative to PIRA, and the consistency across cohorts, despite varying levels of disability progression, may help inform biological/phenotypic categorization in MS. If the same neurodegenerative processes are seen in PIRB compared to those with inactive SPMS/PPMS, it may represent a new avenue in which to focus our future therapeutic efforts.

## Data Availability

The raw data supporting the conclusions of this article will be made available to qualified investigators upon reasonable request, but individual patient data will not be shared.

## Author’s note

The corresponding author CC-L takes full responsibility for the data, the analyses and interpretation, and the conduct of the research; the principal author had full access to all of the data; and has the right to publish any and all data separate and apart from any sponsor.
