# Supplementary material for: Progression independent of relapsing biology in multiple sclerosis: a real-word study
Source: Front Neurol. 2025 May 29;16:1595929. doi: 10.3389/fneur.2025.1595929 (PMC12158712; doi:10.3389/fneur.2025.1595929)
Supplement: Supplementary file 1 [file Table_1.DOCX]

**Supplementary Table 1. Participants excluded due to relapses and/or MRI activity in relation to the timing of disability progression events.**

| **Disability Progression**  **(all DMT populations)** | **Relapses and/or MRI activity** | **Relapses** | **MRI activity** | **Both** |
| --- | --- | --- | --- | --- |
| n=160 | n=73 (45.6%) | n=11 (15.1%) | n=37 (50.7%) | n=25 (34.2%) |
| Within 1yr of first EDSS change | n=55 (75.3%) | n=8 (72.7%) | n=26 (70.3%) | n=21 (84%) |
| >1yr removed from first EDSS change | n=18 (24.7%) | n=3 (27.3%) | n=11 (29.7%) | n=4 (16%) |

DMT=disease modifying therapy; EDSS=expanded disability status scale, yr=year.
